# The Impact of Clinical Training Seminars on Stress and Perception of Clinical Placement Stressors among Spanish Undergraduate Nursing Students: A Two-Phase Mixed-Methods Study

**DOI:** 10.3390/healthcare11030300

**Published:** 2023-01-18

**Authors:** Isabel Lepiani-Díaz, Alberto Paramio, José L. Palazón-Fernández, Daniel Román-Sánchez, María José Carranza-Naval, Concepción Mata-Pérez, Alberto Cruz-Barrientos, Juan Carlos Paramio-Cuevas

**Affiliations:** 1 ‘Salus Infirmorum’ University Nursing Centre, University of Cadiz, 11001 Cadiz, Spain; 2 University Institute of Research in Social Sustainable Development (INDESS), University of Cadiz, 11001 Cadiz, Spain

**Keywords:** clinical placement, nursing students, clinical training seminars, nursing education, perceived stress, mixed-methods study

## Abstract

Stress and stressors related to clinical practice are some of the main reasons for the discomfort reported by nursing students. It is important to identify the causes of stress and seek strategies to reduce the stress levels in nursing students. Clinical training seminars have proven to be a useful tool to reduce stress levels. This study aims to evaluate the effects of a series of clinical training seminars on the levels of stress and perception of stress factors before the start of clinical practice among undergraduate Spanish nursing students. A two-phase, sequential mixed-methods design was used. For the quantitative phase, data were collected using Cohen’s Perceived Stress Scale and the KEZKAK questionnaire before and after the clinical training seminars. Qualitative data were collected through a focus group session held after the clinical training period. The results show a significant reduction (*p* = 0.002) in perceived stress levels after the clinical training seminars, and also a change in students’ perception of stressors in the clinical placement. This study provides valuable information for the development of content for clinical training seminars. Universities should develop strategies to reduce stress in their students caused by the clinical placement.

## 1. Introduction

One of the main aims of universities is to produce highly qualified professionals, regardless of the discipline in which they work. To this end, work placements are essential for students, as they enable them to put into practice the theory learnt in the classroom and acquire new skills, such as the ability to be a part of a team in a real working environment [1,2]. 

The training and experience acquired by students during their work placements are even more important in Health Sciences degrees, where individuals often work in highly sensitive circumstances and conditions with little margin of error. This makes the healthcare environment highly stressful, resulting in higher levels of perceived stress among students in this field than in other areas [3,4,5]. Many studies indicate that Health Sciences students are at risk of experiencing high levels of stress, anxiety and academic burnout, which can negatively affect their academic performance and even their personal lives [6,7]. Nursing students, of course, are not an exception: they must undertake extensive theory and practical training, high expectations are placed on their professional performance, and they are expected to acquire highly complex skills [8]. Some authors [9,10] have indicated that nursing students experience significantly more stress and stress-related outcomes than other non-nursing students and, among them, second-year students report the highest levels of stress due to their perception of having inadequate knowledge and skills in their clinical rotations [11,12]. Stressors affecting nursing students during their clinical placement include uncertainty, a lack of professional knowledge, fear of committing medical errors, performance stress related to expectations from academic and clinical staff, exposure to the death and social problems of patients, and dealing with emergencies [1,13,14,15,16,17,18]. In order to understand this phenomenon, it is crucial to identify the situations perceived by students as stressful during their clinical placements and, more importantly, to do so before students embark on their placements, as high levels of stress can affect their adjustment to the healthcare environment and have a negative influence on their training and health [1], leading, in some cases, to academic burnout [7]. Several studies [1,7,12,19,20,21,22,23] have suggested the importance of actively developing and implementing intervention programs to reduce stress and academic burnout in nursing students. Some studies have shown that simulation in practice learning can be effective in decreasing stress and increasing confidence in clinical practice [24,25], although there are also results opposing this idea [26]. Therefore, other alternatives should be considered in order to meet all the needs of students before the clinical placement.

### 1.1. Background and Setting

The study was conducted at Salus Infirmorum, a Nursing Studies University Center affiliated with the University of Cadiz (Spain). The center is staffed by 18 nursing educators and offers a full-time degree in nursing, consisting of eight academic semesters equivalent to 4 years of university studies. Clinical practice is an important part of the nursing program and provides the students with an opportunity to apply their theoretical knowledge in the clinical placement and, at the same time, to develop individual and clinical skills required for patient care; it also stimulates students’ critical thinking for problem solving. Students take seven subjects on clinical practice, starting in the second semester of the second year, with Practicum I. This subject (12 ECTS) dedicates 196 h to basic patient care, support and monitoring, sample taking and medication administration. The length of the initial clinical practice is two months, with 4 days/week, 7 h/day shifts. The practice takes place in public and private hospitals and primary healthcare settings with learning environments that include several categories of employees, shifts, and patients with challenging medical and nursing requirements. Registered nurses are selected as supervisors; their function is to support students in the learning process and ensure patient safety. Furthermore, students are supported by a faculty professor, who is responsible for their progress and aim achievement. In Spain, the initial clinical experience has long been recognized as the most stressful and a major domain of risk to nursing students in comparison to the personal, academic, and social domains [11,20]. Over the last few years, the signs of uncertainty expressed by our students were collected by the lecturers of the degree course in Nursing at the center in an attempt to make the start of their placements as stress-free as possible. Among the most important sources of stress mentioned by our students were “a lack of knowledge and skills” and “fear of making mistakes”. After conducting a review of the published literature on the subject, it was decided to develop a set of theoretical and practical clinical training seminars to enhance students’ self-confidence and decrease their stress levels during their first clinical practices. The seminar program involved not only the personnel of the nursing department but also the healthcare personnel working at the health centers, so that the students who were to start their clinical training at these facilities could acquire the necessary knowledge and skills while feeling more self-confident and less stressed.

### 1.2. Objectives

The main objective of this research was to evaluate the effects of several clinical training seminars on the levels of stress and perception of stress factors before the start of the clinical practice among undergraduate Spanish nursing students. The specific objectives were: (1) to identify potential clinical practice stressors before and after implementing the clinical training seminars; (2) to assess whether the clinical training seminars reduce perceived stress levels associated with clinical placement and change the student’s perception of clinical placement stressors; (3) to measure the correlation between perceived stress levels and clinical placement stressors, and (4) to gain an understanding of the experience of nursing students during their first clinical placement.

## 2. Materials and Methods

### 2.1. Design

An exploratory, two-phase, sequential mixed-methods design was used in which quantitative and qualitative data were collected sequentially and then the results were analyzed jointly. For the quantitative phase, a quasi-experimental pre–post design was used, taking the clinical training seminars as the intervention point. Three days of clinical training seminars, with a duration of three hours each, were scheduled in the week prior to the start of the clinical placements (Table 1). For the sessions, the participants were divided into four groups of 16 people each. 

For the subsequent qualitative phase, after completing their first clinical placement, a focus group discussion was conducted by a moderator to supplement the quantitative results, exploring in depth the students’ perspectives and opinions about the stressors in clinical placements, and whether the clinical training seminars helped them to reduce their stress levels prior to their first clinical placement. 

### 2.2. Sample/Participants

The study was conducted in 2019. For the quantitative phase, no sampling was used since the study attempted to cover the whole population (n = 71) of students registered for Practicum I. The inclusion criterion was being a full-time second-year undergraduate nursing student at Salus Infirmorum, University of Cadiz (Spain), without previous experience in clinical practice, enrolled in Practicum I in the 2018–2019 academic year, and expressing written consent to participate in the study. The only exclusion criterion was “currently receiving psychological treatment and medication”, because this could influence the perception of stress. In total, 64 students (90.1% of the total) gave their consent and were included in the study. Participants for the qualitative phase (focus group discussion) were selected using a convenience non-probabilistic sample of 12 of the above-mentioned students.

### 2.3. Data Collection and Instruments

Quantitative data for the study were collected in April 2019, at two different points in time, before and after the clinical training seminars, within a two-week interval. The data were recorded manually for further analysis. A paper-based questionnaire packet was administered to each participant and consisted of the following:

- Ad hoc demographic questionnaire. It contained questions regarding age, gender, composition of the household, and place of residence, used to characterize the group. It also asked about the participant’s history of mental health, including treatment and medication.
- The Spanish adaptation of Cohen’s Perceived Stress Scale (PSS) [27,28,29]. The Perceived Stress Scale is an instrument used to assess the degree to which situations in one’s life are appraised as stressful. The scale consists of 14 items scored on a 5-point Likert scale ranging from 0 to 4 (0 = never, 1 = rarely, 2 = sometimes, 3 = often, 4 = very often). Scores range from 0 to 56 points, with higher scores indicating greater perceived stress. The European Spanish version of PSS demonstrated adequate reliability (internal consistency, α = 0.81, and test–retest, r = 0.73), validity (concurrent), and sensitivity [29].
- Clinical placement stressors were assessed using the KEZKAK questionnaire [30]. This questionnaire measures potentially stressful situations during the clinical practice of nursing students. Scores range from 0 to 123, with higher scores indicating greater stress caused by clinical placements. The KEZKAK questionnaire demonstrated adequate reliability (internal consistency, α = 0.95, and test–retest, r = 0.72), validity (concurrent), and sensitivity [30]. The questionnaire consists of 41 items scored on a 4-point Likert scale ranging from 0 to 3 points, quantifying the level of stress caused by the situation described in each item (0 = none, 1 = some, 2 = quite a lot, 3 = a lot). The items are grouped into 9 factors representing potentially stressful situations in student nurses’ clinical placements: Factor 1: Lack of competence (11 items) related to fear of causing harm (to oneself or the patient) or being unable to help the patient; Factor 2: Contact with suffering (10 items); Factor 3: Relationship with tutors and workmates (6 items), related to the relationship with mentors and peers; Factor 4: Helplessness and uncertainty (11 items); Factor 5: Inability to control the relationship with patients (8 items); Factor 6: Emotional involvement (4 items), related to emotional involvement with patients and professional responsibilities; Factor 7: Being harmed by the relationship with the patient (5 items), related to abuse or lack of consideration on the part of the patient and consequent distress; Factor 8: Patients seeking an intimate relationship (2 items), related to behaviors suggesting that the patient seeks an intimate relationship with the student; Factor 9: Overwork (5 items), related to work overload. Based on the total KEZKAK and its factors, a weighted score from 0 to 3 is obtained, dividing the total score by the number of items in the factor, with higher scores indicating higher perceived stress.

Students who gave written consent to participate in the study were asked to fill in the questionnaires. Completion of the three questionnaires required 10–15 min.

A focus group session was held to gain insight into students’ perceptions of their fears and insecurities after their clinical training. The results are presented using a dense description method [31] that allows others to judge the resonance or transferability of the results to other populations and contexts. Participants’ anonymity was preserved by assigning them a number according to their order of participation in the session. The most significant and/or most repeated statements throughout the session were collected and assigned to the various thematic areas identified.

The results were classified, categorized, integrated, and supplemented according to the areas assessed following the methodological guidelines set out by Tashakkori and Teddlie [32]. 

Participants’ contributions in the focus group session were moderated by an external researcher. The focus group session lasted approximately one hour and followed a semi-structured discussion guide (Table 2). The session was conducted in a comfortable environment so that the participants felt relaxed and to encourage group members to share information with their peers and explain their personal feelings concerning the clinical environment. The moderator explained the purpose of the focus group, and asked general questions following the topic guide to provoke and guide the discussion loosely and keep the conversation on track, ensuring that everyone had a chance to speak. The session was recorded and manually transcribed verbatim by the research team.

### 2.4. Data Analysis

The Kolmogorov–Smirnov test for normality was performed, revealing that the data on the quantitative variables were not normally distributed (*p* < 0.05). Descriptive statistics were performed for all parameters, considering the median and interquartile range for quantitative variables and frequencies and percentages for qualitative variables. Comparisons between pre- and post-clinical training seminars were made using Wilcoxon’s test for paired data. Spearman’s correlation coefficient was used to assess the relationship between perceived stress and potential stressors as measured by the KEZKAK questionnaire. Reliability analysis of the scales was performed using Cronbach’s alpha coefficient.

The data were analyzed using IBM’s Statistical Package for the Social Sciences (SPSS) v25 software. The statistical significance threshold for all tests was set at *p* < 0.05. Content and thematic analyses were performed by the external research collaborator who conducted the focus group session, using the qualitative data analysis computer software NVivo by QSR International.

### 2.5. Validity and Reliability/Rigour

In the quantitative phase, the internal consistency of both questionnaires was measured using Cronbach’s alpha (α > 0.88). Corrected item-total correlations were tested using Pearson’s correlation coefficients (r > 0.40). Changes in alpha caused by the removal of each item from the scale were also assessed [33]. As Tracy and Hinrichs [34] suggest, rigor, sincerity, credibility, and resonance denote excellence in qualitative research. These elements were taken into account in the qualitative phase of this study by using an adequate number of participants in the focus group session (n = 12) and by preparing questions for an open-ended interview [35].

## 3. Results

A total of 64 second-year undergraduate nursing students aged 19–25 years (M = 20) participated in the study. The majority of the sample was female (89.1%) and 98.4% lived in different locations in the south of the region of Andalusia (Spain). Most of them were living with their families (67.2%) or in flats shared with other students (17.2%). Moreover, 15.6% reported having received psychological treatment in the past for various reasons.

### 3.1. Perceived Stress

Table 3 shows the results for perceived stress at both points in time. A significant decrease in perceived stress was observed after the clinical training seminars (T = 3.095; *p* = 0.002). After analyzing the PSS items, it was observed that this decrease was caused by an improvement in items 10 (“In the last month, how often did you feel that you had everything under control?”) and 12 (“In the last month, how often have you thought about the things you still have to do?”).

### 3.2. Clinical Placement Stressors

Most of the factors in the KEZKAK questionnaire obtained values above the midpoint of the scale, although these were slightly lower after the clinical training seminars (Table 4). Within the KEZKAK factors, significant differences were observed between the pre- and post-test for Factors 2 (“Contact with suffering”), 4 (“Helplessness and uncertainty”), 5 (“Lack of control over the relationship with the patient”), and 6 (“Emotional involvement”). In all cases, the scores decreased after the clinical training seminars. The factors perceived as most stressful (with the greatest standardized difference in relation to their scale mean) before the seminars were Factors 4 (“Helplessness and uncertainty”) and 5 (“Lack of control over the relationship with the patient”). After the seminars, however, the greatest standardized difference in relation to the scale mean was found in Factor 4 (“Helplessness and uncertainty”). The least stressful factors at both points in time were Factors 3 (“Relationship with mentors and peers”) and 8 (“The patient seeks an intimate relationship”).

### 3.3. Correlations between Perceived Stress and Scores of the KEZKAK Scale

As shown in Table 5, significant correlations were observed between stressors and perceived stress. Before the seminars, the highest correlation between perceived stress and stressors was found in Factor 4 (“Helplessness and uncertainty”). However, after the seminars, the highest correlation between them was found in Factor 5 (“Lack of control over the relationship with the patient”).

### 3.4. Qualitative Results

The most significant statements obtained during the focus group session are presented below. Each participant’s contributions were structured based on the open-ended interview questions proposed by the researchers. The resulting categorization into thematic areas was used to carry out the content analysis and recommendations (Table 6).

#### 3.4.1. Lack of Support and Interest from Coordinators and Care Units

The most significant statements regarding the students’ relationships with the placement coordinators and their experiences in the various care units were as follows: 12—“Coming across people who don’t want to teach you kind of discourages you from going back there”; 1—“It pretty much depends on us, as well as on the professional you’re assigned to”; 10—“In my case, my coordinator wouldn’t come to check on us… You just shadow whatever nurses are there to get the overall picture.” 

#### 3.4.2. Lack of Confidence in Their Coping and Communication Skills

Many of the students felt insecure about their ability to adjust to their clinical training and the lack of tools for effective communication in the hospital setting: 8—“Everything I’ve learned at uni is fine, but when you have to actually be there (she slaps her hand for emphasis) and do it yourself, it’s completely different”; 6—“You come to the realization that, well, if they see that you’re really interested and willing to learn, then the health worker will… trust you a little bit more”; 4—“I was scared before I came here because I thought I wouldn’t be able to deal with some of the situations I’d face… but the truth is that people here are very welcoming, they tell you ‘come on, give it a go, just do it, it’s okay, you’ll learn eventually, it’s okay if you don’t succeed, you will next time, and the truth is that I’m very happy”.

#### 3.4.3. Fear of Making a Mistake or Not Being up to the Task

Overall, fear and feelings of inadequacy were present in many of the students’ contributions. The following were the most significant statements in this respect: 7—“Well, to be honest, I was very, very scared before starting my placement”; 4—“The [seminars] have definitely helped me because although they didn’t take away the fear of harming the patient completely, they made me feel a bit calmer because they made it all clear to me, all the things I had to do on those days, the days before starting the placement”; 5—“I wasn’t too scared, but I was worried something might happen to the other person if I applied a technique the wrong way or something…”.

#### 3.4.4. Experience in Real-Life Scenarios 

Most students agreed on the importance of introducing more realistic scenarios before starting their clinical training, as well as reviewing basic competencies and skills required for this training. 12—“There’s a big difference between working at a primary care center and working at a hospital…”; 5—“I think (the seminars) were good for us as we could go over things, because the truth is that we were a bit lost. We’d done it all before, but reviewing it never hurts, and things were a bit fresher in our minds when we arrived at our placements”; 5—“They place a lot of emphasis on communication with people, but it’s also true that we could use a bit of… reality too, you know?”.

## 4. Discussion

Similar to other studies in Spain [4,13,14,17,18,19,20,21,36], our nursing students presented moderately high levels of stress at the beginning of their clinical placements, where a “lack of competence”, “contact with suffering”, “helplessness and uncertainty”, and a “lack of control over the relationship with the patient” were the factors perceived as most stressful before the clinical placement. As found by Zupiria et al. [13] and López and López [19], in the present study, the factor “relationship with mentors and peers” was perceived as the least stressful.

Although the clinical training seminars offered at the center did not eliminate perceived stress among the nursing students during their first clinical placements, they significantly reduced the stress and modified students’ perceptions of the factors considered by them as the most stressful. After the intervention, there was a significant decrease in perceived stress, in the total score for the KEZKAK questionnaire, and in the factors “Contact with suffering”, “Helplessness and uncertainty”, “Lack of control over the relationship with the patient”, and “Emotional involvement”. These results are similar to those obtained when some mentoring programs [37] and simulations in practice [25] were implemented in internships. Simulations have proven to be an effective strategy for reducing anxiety and increasing self-confidence, but results are still inconclusive for stress [38]. 

Regarding the correlations between stress levels and the potentially stressful factors in the KEZKAK questionnaire, it was initially observed that the factors “Helplessness and uncertainty”, “Emotional involvement”, and “Relationship with mentors and peers”, in this order, had the strongest correlations with perceived stress levels. However, after the clinical training seminars, the factors “Lack of control over the relationship with the patient”, followed by “Contact with suffering” and “Emotional involvement”, exhibited the strongest correlations with perceived stress. It is relevant to note that the factor with the strongest correlation with perceived stress before the pre-practicum clinical training seminars, “Helplessness and uncertainty”, was not among the three factors with the strongest correlations with perceived stress after the clinical training seminars. As stated by Cheon and You [39], witnessing the death and dying of patients is a strong, often overwhelming experience during clinical practice and it would be necessary to include programs covering aspects such as death and suffering and coping strategies. 

The teaching content of the clinical training seminars held before students’ placements covered the following elements: a review of the main basic nursing techniques; safety in the healthcare environment; and an orientation lecture delivered by the coordinators of the various healthcare facilities on the different departments where the students would carry out their clinical placements. Based on this teaching content, it is clear that the clinical training seminars helped students to control their overall levels of perceived stress prior to their clinical placements, as well as their levels of perceived stress caused by the stressor “Helplessness and uncertainty”, which was described as the most relevant stressor prior to the seminars and may be related to the technical skills and information available prior to the start of the clinical placements. However, according to the results of the clinical training seminars, “Lack of control over the relationships with the patient” appears to be the most important stressor. No activities relating to interpersonal skills in healthcare settings were included in the teaching content for the clinical training seminars. This is directly related to the results obtained in the qualitative part of this study, where a group of students were asked to discuss how the pre-practicum clinical training seminars helped them to cope with the stress of starting their clinical placements.

After analyzing the results, it was observed that the levels of total anxiety decreased significantly after the scheduled clinical training seminars, and students’ perceptions of the main types of stressors in clinical placements changed completely. From this point on, feelings of “helplessness and uncertainty” appear to have been reduced thanks to the teaching content, and factors relating to dealing directly with the world of healthcare became more prominent.

This work highlights the relevance of preparing undergraduate nursing students for their clinical training by implementing seminars that provide a better transition between theoretical and practical training. Although the seminars implemented in our study were effective in achieving the objectives set, we recommend that further studies with seminars should be conducted to address all the needs that may arise from the students in order to achieve their satisfaction with the training received.

## 5. Limitations

This research was conducted with students enrolled in the Practicum I module from a single institution, limiting the generalizability of the results. Nursing students in other universities in Spain and in other countries may have a different experience of stressors during their clinical placement. Since all data were collected through self-administered questionnaires, the study was prone to response bias. Nonetheless, the results obtained are highly promising for the design of new clinical training seminars aiming to improve the education of future nursing professionals, facilitate their incorporation into the healthcare system, and transform clinical placements into positive, satisfactory experiences. We recommend including, as part of the pre-clinical training seminars, one or more sessions devoted to stress management techniques to help students to cope with the stress caused by the clinical placement, with a specific emphasis on nurse–patient relationships and witnessing the death and suffering of the patients and their families.

## 6. Conclusions

After completing the study and analyzing the results, it is concluded that the pre-practicum clinical training seminars implemented helped students to adapt to the world of healthcare as part of the Practicum I module, significantly reducing their perceived stress levels. Once students had attended the seminars, their concerns shifted to factors relating to “Lack of control over the relationship with the patient”. This finding was reinforced by the results obtained from the focus group analysis, where students stressed the importance of designing seminars to focus on real-life scenarios or based on real-life experiences, with specific training adapted to the type of care to be provided at the different healthcare facilities before starting their placements. The seminars were perceived by the students as very useful in preparing them for clinical training. 

In conclusion, the results of this study provide us with valuable information to refine the content of these seminars by focusing on more practical aspects that help students to reduce their stress levels before their first experiences with their clinical placements. Reduced stress levels will lead to better performance during the clinical placement, facilitating learning and enabling better incorporation into the healthcare workplace. It is therefore recommended that seminars be held prior to clinical placements, focusing on improving communication skills with patients, increasing confidence with nursing techniques and instruments, and introducing students into more similar settings to those that they will encounter in their clinical training.

## Figures and Tables

**Table 1:** Pre-placement training seminar schedule for Practicum I.

	Day 1	
Time	Topic	Group
10:00–11:00	- Presentation and clinical placement regulations	Whole Group
	- Basic patient care (hygiene and mobilization)	Group 1
	- Basic patient support (vital signs and EKG)	Group 2
11:00–12:30	- Electrolyte monitoring (nutrition and elimination)	Group 3
	- Sample taking and medication administration safety	Group 4
	- Basic patient care (hygiene and mobilization)	Group 2
	- Basic patient support (vital signs and EKG)	Group 3
12:30–14:00	- Electrolyte monitoring (nutrition and elimination)	Group 4
	- Sample taking and medication administration safety	Group 1
	Day 2	
Time	Topic	Group
10:00–11:30	- Basic patient care (hygiene and mobilization)	Group 3
	- Basic patient support (vital signs and EKG)	Group 4
	- Electrolyte monitoring (nutrition and elimination)	Group 1
	- Sample taking and medication administration safety	Group 2
11:30–13:00	- Basic patient care (hygiene and mobilization)	Group 4
	- Basic patient support (vital signs and EKG)	Group 1
	- Electrolyte monitoring (nutrition and elimination)	Group 2
	- Sample taking and medication administration safety	Group 3
	Day 3	
Time	Topic	Group
10:00–11:30	- Presentation and reception, San Juan Grande Healthcare Facility regulations	Whole Group
11:30–13:00	- Protocol for action in the event of biological accidents	Whole Group

**Table 2:** Examples of the questions used to guide the focus group discussion.

- Please briefly introduce yourself. - How do you feel about the clinical placement so far? - What did you worry about regarding clinical experiences? - Was there anything in particular that caused you emotional stress during your clinical practice? - Would you like to talk about those clinical experiences which you found most stressing? - How did this affect you? - Before you started your clinical practice, what were your expectations? - You attended a series of clinical training seminars. What is your opinion about them? did you find them useful for your clinical practice? - Did the clinical training seminars change your expectations about the clinical practice? - Do you think the training seminars helped to reduce your stress levels before you start the clinical practice? - What part of the clinical training seminars resulted more helpful for you? - How do you think clinical experiences can be improved? - Is there anything you would change or add to the clinical training seminars? - Do you have anything else to say?

**Table 3:** Univariate descriptors of the Perceived Stress Scale items before and after the clinical training seminars.

Item	Pre *M* (IQR)	Post *M* (IQR)	Wilcoxon–Mann–Whitney	
			*T*	*p*
01	2.00 (1)	2.00 (1)	−0.323	0.747
02	2.00 (2)	2.00 (2)	−0.551	0.582
03	3.00 (2)	3.00 (1)	−1.919	0.055
04	2.00 (1)	1.00 (1)	−1.469	0.142
05	1.50 (1)	1.00 (1)	−0.465	0.642
06	2.00 (1)	2.00 (1)	−0.194	0.846
07	2.00 (1)	2.00 (1)	−1.892	0.058
08	2.00 (2)	2.00 (2)	−0.676	0.499
09	1.00 (1)	1.00 (1)	−1.608	0.108
10	2.00 (2)	2.00 (1)	−2.256 *	0.024 *
11	2.00 (2)	2.00 (2)	−1859	0.063
12	4.00 (1)	3.00 (2)	−4.123	0.000 *
13	1.00 (1)	2.00 (1)	−1.318	0.188
14	2.00 (2)	2.00 (2)	−1.829	0.067
Total	28.00 (13)	27.00 (12.75)	−3.095	0.002 *

* Sig = *p* ≤ 0.05.

**Table 4:** KEZKAK clinical placement stressors before and after the clinical training seminars.

Factor	Pre *M* (IQR)	Post *M* (IQR)	*p*
Lack of competence	2.09 (0.70)	1.91 (0.73)	0.069
Contact with suffering	2.10 (0.75)	1.95 (0.70)	0.009 **
Relationship with mentors and peers	1.75 (0.83)	1.67 (1.00)	0.191
Helplessness and uncertainty	2.36 (0.73)	2.09 (0.82)	0.000 **
Inability to control the relationship with the patient	2.12 (0.75)	1.88 (0.75)	0.000 **
Emotional involvement	2.00 (1.00)	1.75 (0.75)	0.000 **
Being harmed by the relationship with the patient	1.80 (1.15)	1.80 (0.80)	0.348
Patient seeking an intimate relationship	2.00 (1.00)	2.00 (1.5)	0.457
Work overload	1.90 (0.80)	1.80 (0.80)	0.127
Total	1.95 (0.72)	1.85 (0.73)	0.021 *

* Sig = *p* ≤ 0.05; ** Sig = *p* ≤ 0.01.

**Table 5:** Spearman’s correlation coefficients between pre- and post-test for perceived stress and KEZKAK clinical placement stressors.

KEZKAK Factor	PSS	
	Pre	Post
Lack of competence	0.145	0.236
Contact with suffering	0.233	0.395 **
Relationship with mentors and peers	0.254 *	0.265 *
Helplessness and uncertainty	0.302 *	0.357 **
Inability to control the relationship with the patient	0.197	0.431 **
Emotional involvement	0.291 *	0.359 **
Being harmed by the relationship with the patient	0.040	0.177
Patient seeking an intimate relationship	0.136	0.213
Work overload	0.224	0.330 **

* Sig = *p* ≤ 0.05; ** Sig = *p* ≤ 0.01.

**Table 6:** Focus group content analysis and recommendations for improvement.

Themes	Content
Lack of support and interest from coordinators and care units.	In the focus group session held during the clinical training, students stated that their perceptions changed according to their participation in the care activities carried out in each workplace and the involvement of their mentors in their training. This was an important, recurring theme in the focus group, as students stated that many health professionals are not willing to invest their time in teaching techniques and tasks to trainees. Discussions with coordinators received the most negative feedback, as students did not feel that coordinators and mentors took responsibility for them or were involved in their training. They felt that the support that they received from other nurses was essential for their training and for their work placements to be a positive experience.
Lack of confidence in their coping and communication skills.	Students agreed on the need to improve communication skills with patients and families, especially in critical situations: communicating the death of a patient to family members, explaining a complex procedure, etc.
Fear of making a mistake or not being up to the task.	Most students stated that the clinical training seminars would never fully prepare them for the reality of work. However, they were grateful for the refresher training, although many did not explicitly state that the seminars constituted a protective factor against stress. Fear cannot be completely eliminated, but it is possible to increase confidence. Most students found the seminars to be an essential tool. Reinforcing knowledge and technical procedures prior to actual clinical training helps students to build confidence in care settings while promoting the development of a professional vocation. The clinical training seminars were highly rated for their training in patient care techniques and for their activities designed to teach basic care delivery skills.
Experience in real-life scenarios.	The students reported considerable differences between the various facilities where they carried out their clinical placements, especially between primary care centers and public hospitals. The short time spent by nurses in the facilities during their training was highlighted as one of the main causes of demotivation, along with a low level of involvement in patient care and treatment. Prior clinical training experience is the main reducer of stress before commencing the placement.
Recommendations for Improvement	
Students agreed that clinical placements are not inherently difficult, but stated that their adaptation and involvement in each setting depend largely on external factors.	
In order to improve the clinical training seminars, students suggested increasing the number of clinical cases that realistically represent the situations that they will face during their clinical placements and tailoring them to the specific type of facility that they will be working at (primary care center or public hospital). Above all, they proposed the inclusion of a greater number of activities designed to improve communication skills with patients and families, which they consider to be essential for their training. Students said that the clinical training seminars allowed them to start their placements better prepared and more self-confident than students from other educational centers and were hopeful that the seminars would continue to be offered in the future.	
In addition, all students agreed on the importance of their initial training in confirming their decision to study nursing and their commitment to the discipline, which is why such training could be seen as a catalyst for a professional vocation. This further emphasizes the importance of improving students’ experiences and integration into their clinical placement settings.	

## Data Availability

The data presented in this study are available on request from the corresponding author.
